# Comparison of efficacy and safety of combined phacoemulsification, goniosynechialysis and goniotomy with trabeculectomy in advanced primary angle-closure glaucoma: a retrospective observational study

**DOI:** 10.3389/fmed.2025.1581356

**Published:** 2025-07-23

**Authors:** Wenzhe Zhang, Pengyun Wang, Chen Wang, Wenhui Liu, Jiayin Wu, Lin Shen, Rachita Kurmi, Hui Guo

**Affiliations:** ^1^Department of Ophthalmology, Shandong University, Jinan, Shandong, China; ^2^Ophthalmology Department, School of Basic Medical Sciences, Cheeloo College of Medicine, Shandong University, Jinan, Shandong, China

**Keywords:** primary angle-closure glaucoma, goniosynechialysis, goniotomy, minimally invasive glaucoma surgery, phacoemulsification and intraocular lens implantation

## Abstract

**Purpose:**

The study is a retrospective observational study. This study aimed to compare the intraocular pressure (IOP) lowering effect of phacoemulsification combined with intraocular lens implantation (PEI), Goniosynechialysis (GSL), and goniotomy (GT) vs. trabeculectomy (TRAB) in eyes with medically uncontrolled advanced primary angle-closure glaucoma (PACG) at a 12-month follow-up.

**Methods:**

Patients with medically uncontrolled advanced PACG with 360° peripheral anterior synechia (PAS) were included in this study. The patients were divided into two groups based on the surgical technique: PEI + GSL + GT and TRAB. Each patient completed a 12-month postoperative follow-up.

**Results:**

A total of 73 eyes from 71 patients were included in this study, 43 eyes of which received PEI + GSL + GT and 30 eyes of which received TRAB. At 12 months follow-up, the mean preoperative and postoperative IOP was 27.63 ± 11.38 and 16.46 ± 4.04 mmHg in the PEI + GSL + GT group (*P* < 0.001), and 32.83 ± 13.91 and 15.21 ± 2.58 mmHg in the TRAB group (*P* < 0.001), respectively. As for success rates, among the 43 eyes in the PEI + GSL + GT group, 35 eyes (81.40%) and 40 eyes (93.02%) achieved complete and qualified success with IOP ≤21 mmHg. In the TRAB group, out of 30 eyes, 25 eyes (83.33%) and 29 eyes (96.67%) achieved complete and qualified success with IOP ≤21 mmHg, respectively. Among the 43 eyes in the PEI + GSL + GT group, 33 eyes (76.74%) and 37 eyes (86.05%) achieved complete and satisfactory success with IOP ≤18 mmHg, respectively. While in the TRAB group, 24 eyes (80.00%) and 27 eyes (90.00%) achieved complete and qualified success, respectively, with IOP ≤18 mmHg. There was no significant difference in visual field (VF) between preoperative and postoperative periods in both groups. All eyes exhibited no serious postoperative complications that threatened vision.

**Conclusions:**

PEI + GSL + GT and TRAB demonstrated comparable efficacy in lowering IOP, reducing medications, and preserving VF in medically uncontrolled advanced PACG. Moreover, PEI + GSL + GT exhibited fewer complications, avoid bleb-related complications and may be a novel minimally invasive alternative for treating advanced PACG.

## 1 Introduction

Glaucoma is the leading cause of irreversible vision loss worldwide (1). Studies have demonstrated that the overall prevalence of glaucoma in people over 40 years of age in China ranges from 1.5 to 3.6%, with the prevalence of primary angle-closure glaucoma (PACG) ranging from 0.5 to 1.6% (2). Early symptoms of PACG are mostly inconspicuous and difficult to detect. Studies have revealed that 75% of patients with PACG develop into intermediate and advanced glaucoma at their first visit, leading to missed opportunities for timely treatment (3).

Intraocular pressure (IOP) is an important risk factor for the development and progression of glaucoma, and it is also the only factor that can be controlled (4). Trabeculectomy (TRAB) is the gold standard for surgical treatment of glaucoma, as it may reduce IOP quickly and effectively. However, TRAB remains imperfect as it has several serious postoperative complications, including persistent hypotony and bleb-related complications. With the advent of minimally invasive glaucoma surgery (MIGS), it is gradually being promoted by glaucoma surgeons due to its minimally invasive, effective, and safe benefits.

MIGS involves a wide array of surgical techniques and devices that aim to lower IOP with a higher level of safety than traditional glaucoma surgeries, such as Gonioscopy-Assisted Transluminal Trabeculotomy (GATT), the Kahook Dual Blade, iStent, ab interno trabeculotomy and minimally invasive bleb surgeries like Xen Implant. Those ab interno procedures are performed inside the eye and create a new drainage pathway to lower IOP by cutting the inner wall of Schlemm's canal (SC) while preserving the outer wall (5). They can be performed stand-alone or with phacoemulsification combined with intraocular lens implantation (PEI). Goniosynechialysis (GSL) and goniotomy (GT) combined with PEI has appeared to be an effective treatment for PACG (6, 7). PEI alone or combined with GSL has been exhibited to effectively reduce IOP and prevent further progression of early and mid-stage PACG (8–10); however, it is less effective for advanced PACG. Hamanaka et al. (11) found that the function of SC and trabecular meshwork (TM) function is also impaired in the cases of long-term peripheral anterior synechia (PAS), leading to the poor outcome of GSL treatment for advanced PACG. This could be because persistent iris-trabecular contact or PAS may cause a progressive process of SC endothelial damage, subsequent SC occlusion, and trabecular cell damage, possibly due to mitochondrial dysfunction and subsequent fusion of the trabecular beams. In this context, GT serves to lower IOP by directly incising the inner wall of SC, which effectively alleviates the resistance caused by long-term PAS and trabecular dysfunction and enhances the flow of aqueous humor (12, 13). Compared to other MIGS, PEI + GSL + GT could effectively deepen the anterior chamber, resolves PAS and pathological TM, and therefore, enhances the flow of aqueous humor in PACG. Nevertheless, this combined surgical procedure may necessitate a longer recovery period and be prone to specific complications compared with traditional glaucoma surgery TRAB.

Compared to other MIGS, PEI + GSL + GT could effectively deepen the anterior chamber, resolves PAS and pathological TM, and therefore, enhances the flow of aqueous humor in PACG. However, this combined surgical procedure may necessitate a longer recovery period and maybe prone to specific complications compared with traditional glaucoma surgery TRAB.

Most of recent studies which have displayed the efficacy of PEI, GSL and GT for treating PACG were single-arm trials (14–17). Our study aimed to compare the IOP-lowering effect and surgical complications of PEI, GSL and GT vs. TRAB in eyes with medically uncontrolled advanced PACG and hypothesized that the efficacy of the minimally invasive procedure PEI + GSL + GT was non-inferior to TRAB but less invasive and safer. This may provide a new minimally invasive alternative for treating advanced PACG.

## 2 Methods

The study is a retrospective observational study. Participants with PACG who visited our hospital between May 2020 and February 2023 were recruited for this study. This study was approved by the Ethics Committee of our hospital. The study protocol was designed per the Declaration of Helsinki. Written informed consent was obtained from all participants.

The inclusion criteria were as follows: (1) age 40 years and above; (2) IOP >21 mmHg with the maximum-dose use of IOP-lowering medications; (3) PAS: the presence of 360° PAS as observed under gonioscopy; (4) advanced glaucoma: obvious glaucomatous optic neuropathy and glaucomatous visual field (VF) defects; according to the Hodapp-Parrish-Anderson criteria, advanced stages were defined as mean deviation (MD) < -12 dB; and (5) obvious cataract which needs for lens extraction as assessed by a clinician.

The exclusion criteria were as follows: (1) history of ocular trauma and ocular diseases other than glaucoma and cataracts, and (2) other types of glaucoma, including open-angle, secondary angle-closure, steroidal, and angle-regression glaucoma.

### 2.1 Data collection

All patients underwent a complete preoperative ophthalmic examination, including slit lamp examination, slit lamp fundus examination, slit lamp gonioscopy, IOP, best-corrected visual acuity, retinal nerve fiber layer (RNFL) thickness, VF, endothelial cell count, anterior chamber depth (ACD), and axial length (AL). VF examination was performed using a Humphrey VF meter (HFA II, ZEISS, Germany). The RNFL was measured using optical coherence tomography (CIRRUS HD-OCT 4000, ZEISS, Germany). ACD and AL were measured using IOLMaster 700 (ZEISS, Germany). Outcome assessments were conducted by an assessor blind to treatment allocation.

### 2.2 Surgical technique

All procedures were performed by one experienced glaucoma surgeons under general or local anesthesia. The detailed surgical methods are as follows.

For the PEI + GSL + GT procedure, a corneal limbal incision was made at the 3 o'clock position of the cornea and a main incision at the 9–10 o'clock position of the cornea. Then perform cataract phacoemulsification surgery, followed by the implantation of an intraocular lens. After implantation, inject viscoelastic into the anterior chamber. Tilt the microscope ~35° toward the nasal side. With the assistance of an anterior chamber angle mirror, use an iris retractor to gently apply pressure to the nasal and inferior quadrants of the PAS under direct vision, separating the adherent anterior chamber angle until the TM is exposed. At this point, the white wall of SC can be seen, accompanied by a small amount of red reflux. Insert the tip of a 25G needle into the SC in the nasal and inferior quadrants, cutting ~120° of SC and the TM. After aspirating the viscoelastic and blood from the anterior chamber, achieve a watertight corneal incision.

For the TRAB procedure, a conjunctival flap was first created at the limbal region, followed by cauterization of the sclera to control bleeding. Next, a scleral flap, ~3 × 4 mm in size and about half the thickness of the cornea, is prepared. Cotton swabs impregnated with mitomycin (0.4 mg/ml) were placed between the conjunctival flap, scleral flap, and scleral bed for 2.5 min, after which the mitomycin was fully rinsed away using lactated Ringer solution. After removing a small section of trabecular tissue, roughly 1 × 3 mm, a peripheral iridectomy is performed. The superior edge of the scleral flap is secured using two 10-0 nylon sutures along with two adjustable sutures. The conjunctival incision is then closed with 10-0 nylon sutures. Postoperative observations showed that the diffusion of filtrate occurred as anticipated, IOP remained within normal limits, and the surgical procedure was successfully completed.

### 2.3 Outcomes measures

The primary outcome indicator was the postoperative reduction in IOP compared to the preoperative value. Secondary outcome indicators included changes in the number of IOP-lowering medications and the progression of VF damage. Success is defined as an effective reduction IOP (IOP ≤ 21 or ≤ 18 mmHg) without any vision-threatening complications and without the need for additional glaucoma surgical procedures. The success was considered qualified if IOP-lowering medications were needed.

### 2.4 Statistical analysis

The sample size for this study was determined using PASS software. This investigation was designed as a retrospective cohort analysis aimed to determine whether the effectiveness of the combination treatment of PEI + GSL + GT was non-inferior to TRAB. According to existing literature, TRAB typically reduces IOP to an average of 12.4 ± 4.60 mmHg (18). Assuming that the standard deviation of the reduction in IOP in the PEI + GSL + GT study group was also 4.60 mmHg, taking the non-inferiority margin (δ) as 4 mmHg, and setting α = 0.025 (one-sided), the statistical power (1–β) = 0.8. The number of subjects in both groups was equal, and using PASS 2021 software, the sample size for both the treatment group and the control group was calculated to be 22 cases. Assuming a dropout rate of 20% in each group, the minimum required sample size for each group is 22 cases divided by 0.8, which is 22/0.8 = 27.5, rounded to 28 cases. Therefore, each group should have at least 28 cases, totaling no < 56 cases. Ultimately, we collected 43 cases in the PEI + GSL + GT group and 30 cases in the TRAB group, totaling 73 cases.

Data were analyzed using the Statistical Product and Service Solutions (SPSS) software (version 26; IBM Corp.). Measurement data such as age, IOP, type of IOP-lowering medication used, MD of VF, and RNFL thickness are expressed as mean ± standard deviation. Count data, such as gender, history of anti-glaucoma surgery, and surgical complications, are expressed as frequencies and rates.

Before comparing the measurement data, normality and chi-square tests were performed, and if the normal distribution and chi-square were satisfied, the *t*-test was applied. If the normal distribution or variance chi-squared test was unsatisfactory, the corrected *t*-test, Mann–Whitney *U*-test, and Wilcoxon rank-sum test were applied as appropriate. Pearson chi-square and Fisher exact tests were applied to compare the count data. Repeated-measure analysis of variance and paired *t*-tests were used to compare the preoperative and postoperative values within each group. The two groups were compared using an independent sample *t*-test for numerical variables. Kaplan–Meier survival curves were plotted to estimate the mean survival time and probability of failure at different time points. In this study, statistical significance was set at *P* < 0.05.

## 3 Results

A total of 73 eyes from 71 patients were included in this retrospective observational study, 43 eyes of which received PEI + GSL + GT and 30 eyes of which received TRAB. Baseline information of patients between the two groups was not statistically different except for the surgical history (Table 1). Patients in the PEI + GSL + GT group had more previous failed surgeries than those in the TRAB group, indicating that the IOP of PEI + GSL + GT group was more difficult to control than TRAB group. The MD of VF was −23.55 ± 5.92 and −25.29 ± 7.45 dB in the PEI + GSL + GT group and the TRAB group, respectively, indicating that patients of the both group had advanced VF loss.

**Table 1:** Demographic Information and clinical preoperative and postoperative evaluation.

**Characteristics**	**PEI + GSL + GT**	**TRAB**	***P* value**
Participants (no. eyes)	42(43)	29(30)	\
Age (y)	64.09 ± 8.43	66.57 ± 10.85	0.2769
Baseline IOP (mm Hg)	27.63 ± 11.38	32.83 ± 13.91	0.0842
Range of IOP (mm Hg)	7.1–60	12.5–59	\
BCVA (logMAR)	0.26 ± 0.26	0.27 ± 0.26	0.822
ACD (mm)	2.21 ± 0.39	2.46 ± 0.53	0.052
AL (mm)	22.06 ± 3.10	22.65 ± 0.92	0.331
Endothelial cell density (cells/mm^2^)	2495.85 ± 650.53	2662.6 ± 525.38	0.377
C/D	0.77 ± 0.16	0.72 ± 0.21	0.238
Glaucoma medications	2 (1.75, 3)	0 (0, 3)	0.057
RNFL thickness/μm	60.55 ± 13.94	58.4 ± 10.13	0.477
MD of visual field (dB)	−23.55 ± 5.92	−25.29 ± 7.45	0.273
Mean duration of disease (range) (y)	2.61 (0.38–16.44)	2.8 (1–14)	0.828
**Surgical history**			
Yes	30 (69.77%)	4 (13.33%)	0.000^**^
No	13 (30.23%)	26 (86.67%)	
**Gender**			0.153
Female	19 (44.19%)	19 (63.33%)	
Male	24 (55.81%)	11 (36.67%)	

TRAB, trabeculectomy; PEI + GSL + GT, phacoemulsification combined with intraocular lens implantation, goniosynechialysis and goniotomy; IOP, intraocular pressure; BCVA, best-corrected visual acuity; MD, mean defect; PAS, peripheral anterior synechiae; ACD, anterior chamber depth; AL, axial length; RNFL, retinal nerve fiber layer.

*P*-values reflect between-group differences: ^**^P < 0.01.

**Figure 1:**
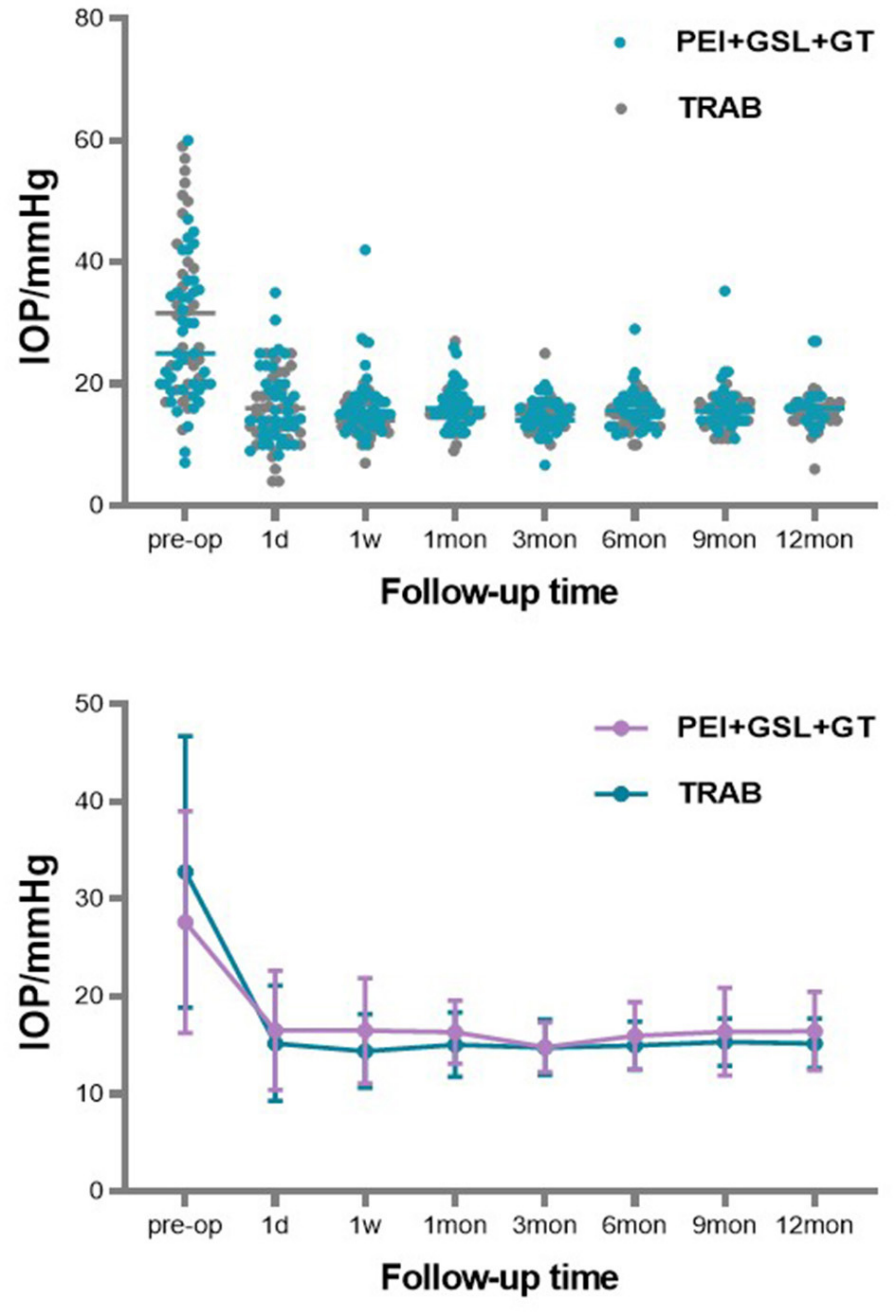
Scattergram of preoperative and postoperative IOP value (on the abscissa). ERROR BAR = SD.

A total of 43 eyes were included in the PEI + GSL + GT group and declined to 20 eyes at 12-month follow-up. Due to poor efficacy or complications, three patients underwent other ophthalmic surgeries. 23 patients discontinued follow-up or were lost to follow-up, in which nine cases lost before the 6-month follow-up, four cases lost before the 9-month follow-up, nine cases lost before the 12-month follow-up, and one case who died for other reasons. To restore the real situation as much as possible and reduce the impact of missing follow-up data on the final results, considering that most missing data was due to patients' stable conditions without subsequent testing, we used the Last Observation Carried Forward (LOCF) method to impute IOP data for the PEI + GSL + GT group, in order to achieve intention-to-treat analysis (ITT). As shown in Table 2 and Figure 1, mean baseline IOP in the PEI + GSL + GT group was 27.63 ± 11.38 mmHg, reduced to 16.52 ± 6.12 mmHg at 1 day postoperation and gradually decreased to a stable level of a mean IOP of 16.46 ± 4.04 mmHg during the 12-month follow-up. The mean IOP at each time point was significantly different from that before surgery (*P* < 0.05). The mean baseline IOP in the TRAB group was 32.83 ± 13.91 mmHg, reduced to 15.20 ± 5.95 mmHg at 1 day postoperation. The surgical effect lasted for 12 months at the level of 15.21 ± 2.58 mmHg and had significant differences at each follow-up time point compared with baseline IOP (*P* < 0.05). There is no significant difference in IOP between the two groups as shown in Table 3 and Figure 1.

**Table 2:** Mean IOP and changes from baseline, and surgical outcomes at each time point.

**PEI + GSL + GT**	**Baseline**	**Day 1 (*n* = 43)**	**Day 7 (*n* = 43)**	**Month 1**	**Month 3**	**Month 6**	**Month 9**	**Month 12**
	**(*****n*** = **43)**			**(*****n*** = **43)**	**(*****n*** = **43)**	**(*****n*** = **43)**	**(*****n*** = **43)**	**(*****n*** = **43)**
IOP (mm Hg)	27.63 ± 11.38	16.52 ± 6.12	16.50 ± 5.39	16.35 ± 3.26	14.80 ± 2.55	15.97 ± 3.45	16.41 ± 4.50	16.46 ± 4.04
*t*		5.642	5.798	6.251	7.219	5.762	5.122	4.255
*P* value	—	0.000^**^	0.000^**^	0.000^**^	0.000^**^	0.000^**^	0.000^**^	0.000^**^
**TRAB**	**Baseline**	**Day 1 (*****n*** = **29)**	**Day 7 (*****n*** = **29)**	**Month 1**	**Month 3**	**Month 6**	**Month 9**	**Month 12**
	**(*****n*** = **30)**			**(*****n*** = **29)**	**(*****n*** = **29)**	**(*****n*** = **29)**	**(*****n*** = **29)**	**(*****n*** = **29)**
IOP (mm Hg)	32.83 ± 13.91	15.20 ± 5.95	14.41 ± 3.77	15.07 ± 3.32	14.79 ± 2.85	14.96 ± 2.49	15.33 ± 2.44	15.21 ± 2.58
*t*		6.382	6.887	6.692	6.845	6.810	6.672	6.709
*P* value	—	0.000^**^	0.000^**^	0.000^**^	0.000^**^	0.000^**^	0.000^**^	0.000^**^

TRAB, trabeculectomy; PEI + GSL + GT, phacoemulsification combined with intraocular lens implantation, goniosynechialysis and goniotomy.

n, number of eyes, P-values reflect between-group differences: ^**^P < 0.01.

**Table 3:** Comparison of postoperative IOP follow-up between PEI + GSL + GT and TRAB.

**Follow-up time**	**IOP/mmHg**		***t***	***P* value**
	**PEI** + **GSL** + **GT**	**TRAB**		
Preoperative	27.63 ± 11.38	32.83 ± 13.91	1.75	0.084
**Postoperative**				
1d	16.52 ± 6.12	15.20 ± 5.95	0.915	0.363
1w	16.50 ± 5.39	14.41 ± 3.77	1.809	0.075
1mon	16.35 ± 3.26	15.07 ± 3.32	1.626	0.142
3mon	14.80 ± 2.55	14.79 ± 2.85	0.018	0.986
6mon	15.97 ± 3.45	14.96 ± 2.49	1.313	0.194
9mon	16.41 ± 4.50	15.33 ± 2.44	1.136	0.261
12mon	16.46 ± 4.04	15.21 ± 2.58	1.322	0.193

TRAB, trabeculectomy; PEI + GSL + GT, phacoemulsification combined with intraocular lens implantation, goniosynechialysis and goniotomy.

The number of IOP-lowering medications decreased in both groups during the follow-up of 12 months as shown in Table 4. The PEI + GSL + GT group used a decreased number of medications from 2 (1, 3) preoperatively to 0 (0, 0) postoperatively and the TRAB group used a decreased number from 1 (0, 3) preoperatively to 0 (0, 1) postoperatively (*P* < 0.01). There was no statistical difference in the number of preoperative medications between the two groups, and there was also no statistical difference postoperatively.

**Table 4:** Medication outcomes.

**Follow-up time**	**PEI + GSL + GT**	**TRAB**	***Z* value**	***P* value**
Preoperative	2 (1, 3)	1 (0, 3)	−1.904	0.057
Postoperative	0 (0, 0)	0 (0, 1)	−0.748	0.455
*Z* value	−6.127	−3.594	——	——
*P* value	0.000^**^	0.000^**^	——	——

TRAB, trabeculectomy; PEI + GSL + GT, phacoemulsification combined with intraocular lens implantation, goniosynechialysis and goniotomy.

P-values reflect between-group differences: ^**^P < 0.01.

We also compared the preoperative and postoperative VF MD changes in two groups and the results showed that patients in the PEI + GSL + GT group had a mean preoperative MD of −23.55 ± 5.92 and a mean MD of −23.37 ± 6.98 postoperatively at 12-month-follow-up, with no statistical difference between the results (*P* = 0.946). Patients in the TRAB group had a mean preoperative MD of −25.29 ± 7.45 and a mean MD of −21.52 ± 9.05 postoperative at 12-month-follow-up, with no statistical difference between the results (*P* = 0.517). There was no significant difference in VF MD between preoperative and postoperative periods in patients who underwent PEI + GSL + GT or TRAB (Table 5).

**Table 5:** VF MD changes.

**Follow-up time**	**PEI + GSL + GT (*n* = 43)**	**TRAB (*n* = 30)**	***t***	***P* value**
Preoperative	−23.55 ± 5.92	−25.29 ± 7.45	1.106	0.273
Postoperative at 12 months	−23.37 ± 6.98	−21.52 ± 9.05	0.344	0.732
*t*	0.067	0.696	——	——
*P* value	0.946	0.517	——	——

TRAB, trabeculectomy; PEI + GSL + GT, phacoemulsification combined with intraocular lens implantation, goniosynechialysis and goniotomy.

For the complications, in the PEI + GSL + GT group, hyphema was seen in eight eyes, corneal edema was observed in three eyes with dense nuclear cataract, IOP spike occurred in three eyes and fibrinous exudate in the anterior chamber occurred in two eyes. The hyphema was self-limited and healed within 1-week postoperation in nearly all cases except for one patient who required anterior chamber washout. The corneal edema recovered in several days after topical steroid treatment. IOP spikes were well-controlled with the withdrawal of steroids, replacement with NSAIDs and supplementary IOP-lowering medications. The fibrinous exudate in the anterior chamber disappeared after enhanced topical steroid treatment. In the TRAB group, two patients developed shallow anterior chamber in early phase and reformed after conservative therapy including compression bandage, cycloplegic agents and enhanced steroids. One eye developed malignant glaucoma requiring secondary surgical treatment (Table 6).

**Table 6:** Complications.

**Postoperative complications (%)**	**PEI + GSL + GT (*n* = 43)**	**TRAB (*n* = 30)**
Hyphema	8 (18.60%)	–
IOP spike	3 (6.98%)	–
Corneal edema	4 (9.30%)	–
Fibrinous exudate	2 (4.65%)	–
Bleb-related complications	–	2 (6.67%)
Malignant glaucoma	–	1 (3.33%)

TRAB, trabeculectomy; PEI + GSL + GT, phacoemulsification combined with intraocular lens implantation, goniosynechialysis and goniotomy.

**Figure 2:**
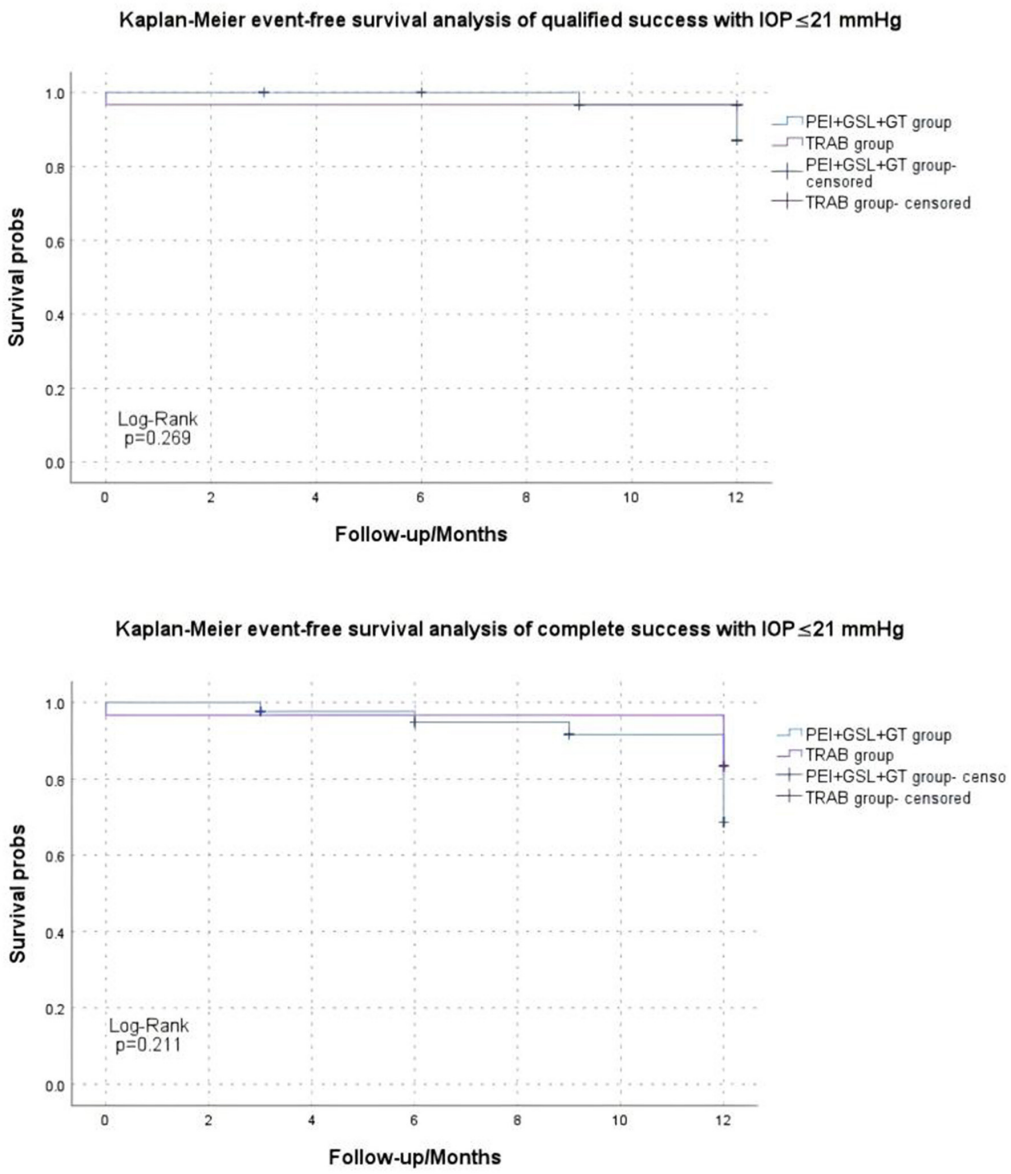
Kaplan–Meier analysis showing the estimated success rates of IOP ≤21 mmHg during the follow-up. “Event” refers to surgical failure (failure to control IOP).

**Figure 3:**
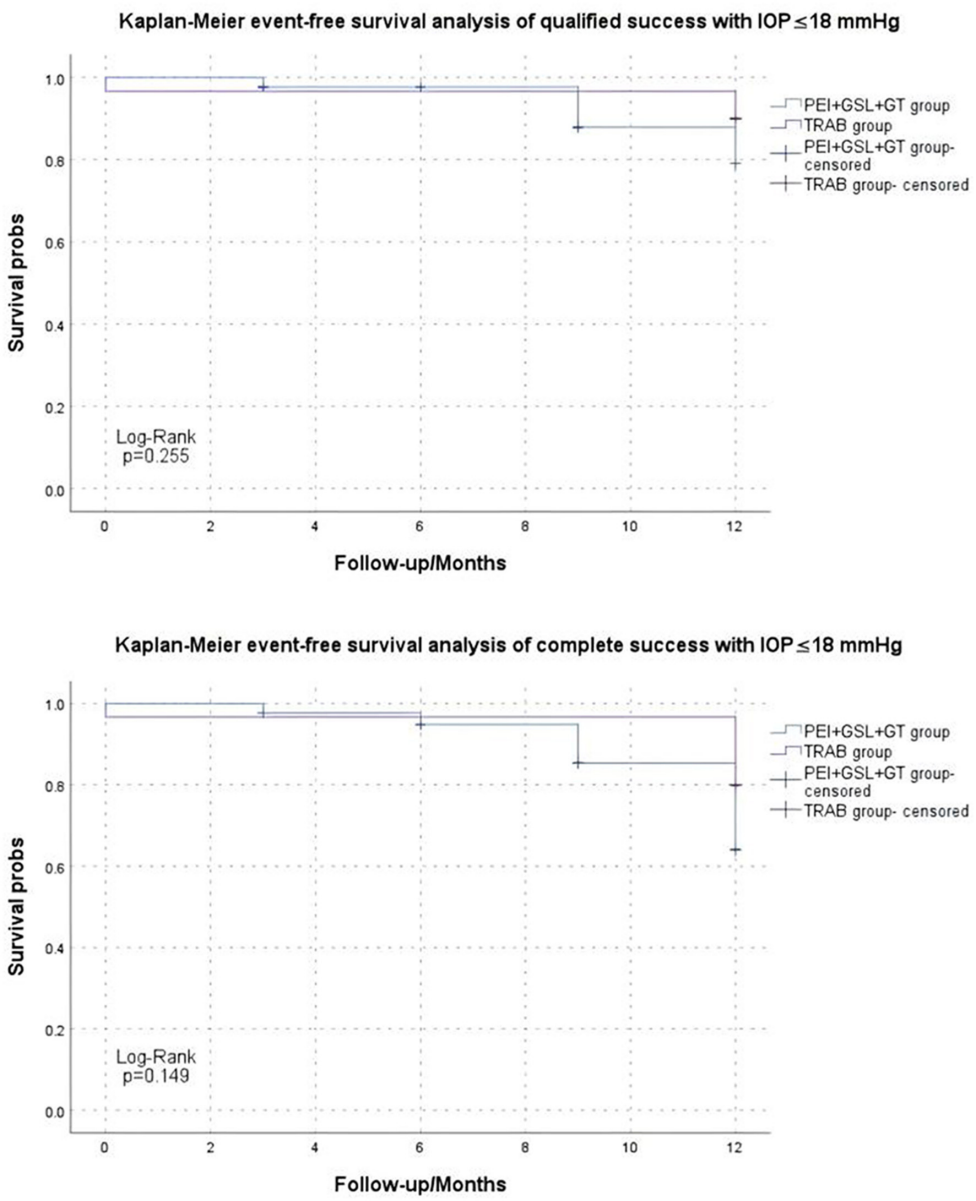
Kaplan–Meier analysis showing the estimated success rates of IOP ≤18 mmHg during the follow-up. “Event” refers to surgical failure (failure to control IOP).

Kaplan–Meier curve summarized the complete and qualified success rates at different follow-up visits (Figures 2, 3). In the case of postoperative IOP ≤ 21 mmHg as the outcome, complete and qualified success was achieved in 35 (81.40%) and 40 (93.02%) of the 43 eyes that received PEI + GSL + GT, and in 25 (83.33%) and 29 (96.67%) of the 30 eyes that received TRAB, respectively. In the case of postoperative IOP ≤ 18 mmHg as the outcome, complete and qualified success was achieved in 33 (76.74%) and 37 (86.05%) of the 43 eyes that received PEI + GSL + GT, and in 24 (80.00%) and 27 (90.00%) of the 30 eyes that received TRAB, respectively (Table 7).

**Table 7:** Success rate (%).

**Success types**	**PEI + GSL + GT (*n* = 43)**	**TRAB (*n* = 30)**
**IOP** ≤ **21mmHg**		
Complete success	35 (81.40%)	25 (83.33%)
Qualified success	40 (93.02%)	29 (96.67%)
**IOP** ≤ **18mmHg**		
Complete success	33 (76.74%)	24 (80.00%)
Qualified success	37 (86.05%)	27 (90.00%)

TRAB, trabeculectomy; PEI + GSL + GT, phacoemulsification combined with intraocular lens implantation, goniosynechialysis and goniotomy.

## 4 Discussion

This study reported the efficacy and safety of PEI + GSL + GT and compared them with TRAB in treating medically uncontrolled advanced PACG with 360° PAS during a 12-month follow-up. The preoperative IOP was similar in both groups, and the IOP was significantly lower in both groups 12 months postoperatively. IOP did not differ significantly between the two groups during the follow-up period, except for the first day and the first week postoperatively. PEI + GSL + GT and TRAB exhibited similar efficacy in lowering IOP and preserving the VF. The success rate at 12 months did not differ significantly between the two procedures. The specific complications varied, with hyphema in the PEI + GSL + GT group and bleb-related issues in the TRAB group being the most common complications.

The IOP-lowering effect of Phaco alone or combined with GSL for early- and mid-stage PACG has been confirmed. However, they appeared less effective for advanced PACG, as SC and TM functions were impaired due to long-term and extensive PAS. Previous studies have reported the efficacy of PEI + GSL combined with GT for treating advanced PACG by incising a certain extent of the TM. However, there is still a lack of consensus on the extent and region of the incising (7, 19). A study conducted by Song et al. (20) showed that a wider incision range may not result in a greater IOP-lowering effect in patients with primary open-angle glaucoma (POAG). Research by Rosenquist et al. (21) reported that the changes in aqueous outflow resistance from 120- to 360-degree trabeculotomy were not significantly different, implying that a Schlemm canal incision >120° provides no additional IOP-lowering effect. In addition, research by Zhang et al. (22) showed that 360-degree GT had the highest incidence of postoperative hyphema, followed by 240-degree GT and 120-degree GT while the three groups did not differ significantly in reducing IOP and medications. Research by Mori et al. (23) suggests that 120-degree incision of the TM and SC showed the fewest complications. These are consistent with our finding that a 120° GT is effective in lowering IOP while also minimizing the risk of complications. In this study, we applied an GT range of 120° in the nasal and inferior quadrants because the collector channels are mainly concentrated in the nasal quadrant, followed by the inferior quadrant (24–26). Removal of the damaged TM may reduce aqueous outflow resistance to the Schlemm canal and ultimately reduce IOP. As we expected, the mean IOP postoperatively during follow-up in the PEI + GSL + GT group reduced significantly (16.52 ± 6.12 mmHg at 1 day postoperation and 16.46 ± 4.04 mmHg at the 12-month-follow-up) compared to the preoperative baseline IOP (27.63 ± 11.38 mmHg). The mean baseline IOP in the TRAB group was 32.83 ± 13.91 mmHg, reduced to 15.20 ± 5.95 mmHg at 1 day postoperation and lasted for 12 months at 15.21 ± 2.58 mmHg, with significant differences at each follow-up time point compared to baseline IOP. The IOP-lowering effect was significantly greater in the TRAB group than in the PEI + GSL + GT group within 1 week postoperatively (*P* < 0.05). However, IOP between the two groups did not differ significantly at subsequent time points after 1 week, which is consistent with the findings of other studies. Dorairaj et al. showed that after 24 months postoperatively, 95.2% of patients who underwent PEI + GSL + GT achieved IOP ≤ 18 mmHg, 100% achieved ≥20% IOP reduction, 85.7% required ≥1 fewer IOP control medications, and 69.0% were medication-free. At 24-month follow-up, no eyes required additional glaucoma surgery (7). Shokoohi-Rad et al. (27) reported that the mean IOP reduction effect was higher in the PEI + GSL + GT group than in the PEI + GSL group (6.93 and 4.6, respectively). Fontana et al. reported that the mean IOP was 30.27 ± 4.20 mmHg preoperatively and 15.20 ± 2.08 mmHg 1 year postoperatively (*P* < 0.001). The mean percentage of IOP reduction from the baseline was 49 ± 9.41%. At 6 and 12 months, the success rates of complete and qualified were 93% (73 and 20%) and 100% (73 and 27%), respectively (28). Therefore, PEI + GSL + GT exhibited good efficacy in lowering IOP in treating advanced PACG.

The number of IOP-lowering medications decreased in both groups during the 12-month follow-up. Patients in the PEI + GSL + GT group used more medications than those in the TRAB group preoperatively, however, the number of medications decreased in both groups during the follow-up of 12 months and there was no significant difference between the two groups. Song et al. (29) reported that the mean preoperative and postoperative IOP at the 1-year follow-up was 27.4 ± 7.3 and 14.2 ± 2.6 mmHg, respectively, and participants used an average of 2.0 and 0.3 types of medications before and after surgery, respectively. Dorairaj et al. (30) reported that the mean number of IOP-lowering medications used was 2.3 (0.1) preoperatively and was reduced at 12 months by 2.2 (0.12), indicating that PEI + GSL + GT has a beneficial effect on reducing IOP and IOP-lowering medications.

We also compared preoperative and postoperative VF MD between the two groups. The results displayed no significant differences in VF MD between the preoperative and postoperative periods in the patients who underwent PEI + GSL + GT or TRAB. This indicated that PEI + GSL + GT and TRAB have a good effect on preserving the VF in patients with advanced PACG.

No severe or vision-threatening complications occurred in either group. One eye in the TRAB group developed malignant glaucoma and received secondary surgical treatment. Malignant glaucoma is one of the most serious complications which in the natural course results in irreversible loss of vision in a short period of time. The risk of malignant glaucoma is associated with penetrating characteristic of glaucoma surgery and its occurrence is higher in eyes with shallow iridocorneal angle and short axial length (31, 32). In contrast, the most common complication of PEI + GSL + GT was hyphema, observed in eight eyes (18.60%), which was mostly self-limiting and recovered within 7 days, except for one patient who required an anterior chamber washout. This is also consistent with previous studies, which reported that the occurrence of hyphema has no impact on the success rate of the surgery (7, 15–17, 19, 27–29, 33–35). Transient high IOP occurred in three eyes (6.98%), and fibrinous exudate in the anterior chamber occurred in two eye (4.65%) after PEI + GSL + GT. Transient postoperative IOP elevation may be due to residual viscoelastic or clot in the anterior chamber. It may also be related to postoperative corticosteroids, which were used postoperatively in all patients in this study. Previous studies on MIGS reported that eyes treated with corticosteroids postoperatively were more likely to have IOP spikes as a complication than eyes treated with NSAIDs only postoperatively. Researchers suggested that there may be a corticosteroid-mediated outflow obstruction in the distal TM (36, 37). However, this conclusion was observed in younger patients with primary open-angle glaucoma after gonioscope-assisted transluminal trabeculotomy, and it remains questionable whether it holds in middle-aged and older PACG patients. Compared to TRAB, PEI + GSL + GT causes fewer complications which could resolve without much intervention and appeared to be less invasive and safer.

In the case of postoperative IOP ≤ 21 mmHg as the outcome, complete and qualified success was achieved in 35 (81.40%) and 40 (93.02%) of the 43 eyes that received PEI + GSL + GT, and in 25 (83.33%) and 29 (96.67%) of the 30 eyes that received TRAB, respectively. In the case of postoperative IOP ≤ 18 mmHg as the outcome, complete and qualified success was achieved in 33 (76.74%) and 37 (86.05%) of the 43 eyes that received PEI + GSL + GT, and in 24 (80.00%) and 27 (90.00%) of the 30 eyes that received TRAB, respectively. This result is similar to that reported in previous studies. Song et al. (29) reported that the complete surgical success rate of PEI + GSL + GT was 89.1%, and the qualified success rate was 95.2%.

In our study, the PEI + GSL + GT and TRAB groups presented promising results in lowering IOP and reducing antiglaucoma medications. At the 12-month follow-up, the PEI + GSL + GT group demonstrated good efficacy in lowering IOP, reducing glaucoma medications, and preserving VF in patients with advanced PACG, similar to the TRAB group. However, PEI + GSL + GT surgery displayed advantages over TRAB since it is minimally invasive, does not need conjunctival incision, and has no bleb-related complications, which may help reduce postoperative ocular surface discomfort and improve the life quality of patients.

In this study, the PEI + GSL + GT group appeared to include more patients with previous surgical failures and one possible reason maybe due to that surgeons tend to employ novel approaches for complex cases. Alternatively, the TRAB group included more patients with limited surgical history since TRAB represents the standard treatment. After adjusting for baseline differences in prior surgical history by detailed preoperative assessment and multivariable analysis (shown in the Supplementary Table 1), our study found that PEI + GSL + GT demonstrated non-inferior efficacy and better safety compared to traditional TRAB in the treatment of advanced PACG, particularly in patients with prior failed filtering surgery. PEI + GSL + GT showed comparable IOP-lowering effects to TRAB and superior safety, suggesting that PEI + GSL + GT may be a valuable treatment option for such complex cases, but further validation is needed through larger-scale studies.

This study has several limitations: (1) We only compared PEI + GSL + GT to TRAB, not PEI + TRAB. We chose to compare with TRAB for two reasons: first, TRAB is well-established as the gold standard for surgical treatment of glaucoma; second, previous studies revealed that both PEI + TRAB and TRAB had comparable long-term IOP-lowering effects with PEI + TRAB having fewer follow-up interventions compared to TRAB (38–40). However, the comparative study over PEI alone or PEI + TRAB will be conducted in future work. (2) The subjects of this study were all Chinese patients; therefore, it cannot represent all PACG patients from different populations. (3) The follow-up period was short. A longer follow-up period is needed to further assess the long-term efficacy of PEI + GSL + GT in the treatment of advanced PACG, including long-term VF preservation and the occurrence of late potential complications. (4) The study was a single-center, retrospective case analysis with a small sample size. There remained a likelihood of encountering potential complications such as a shallow anterior chamber or malignant glaucoma in long-term follow-up (41). A prospective, multi-center, large-sample randomized controlled trial is needed to further evaluate the efficacy and potential complications of PEI + GSL + GT in treating advanced PACG.

## 5 Conclusion

PEI + GSL + GT and TRAB had comparable efficacy in lowering IOP, reducing IOP-lowering medications and preserving VF in medically uncontrolled advanced PACG. PEI + GSL + GT surgery exhibited advantages over TRAB by reducing complications, avoiding bleb-related complications and improving patient life quality, which may become a new option for treating advanced PACG especially for those with previous failed anti-glaucoma surgeries. Further studies are needed to further assess the long-term efficacy and identify risk factors preoperatively to help clinicians provide personalized and precise treatment.

## Data Availability

The raw data supporting the conclusions of this article will be made available by the authors, without undue reservation.
