# Supplementary material for: Comparison of efficacy and safety of combined phacoemulsification, goniosynechialysis and goniotomy with trabeculectomy in advanced primary angle-closure glaucoma: a retrospective observational study
Source: Front Med (Lausanne). 2025 Jul 23;12:1581356. doi: 10.3389/fmed.2025.1581356 (PMC12325217; doi:10.3389/fmed.2025.1581356)
Supplement: Supplementary file 1 [file Table_1.DOCX]

| Table. Multivariate Logistic Regression Analysis for the Factors Associated With Surgical Success | | | | | | | | | |
| --- | --- | --- | --- | --- | --- | --- | --- | --- | --- |
|  | *B* | *SE* | *Wald statistic* | *P* | | | | *OR* | *95% CI* |
| Baseline IOP (mm Hg) | -0.011 | 0.028 | 0.151 | | 0.698 | | 0.989 | | 0.935-1.046 |
| Preoperative glaucoma medications | -0.037 | 0.301 | 0.015 | | 0.902 | | 0.964 | | 0.534-1.738 |
| Surgical History | 0.576 | 0.767 | 0.564 | | 0.453 | | 1.779 | | 0.396-8 |
| IOP, intraocular pressure; OR, odds ratio. | | | | | | |  |  |  |
